# Supplementary material for: Development of Quantitative Chemical Ionization Using Gas Chromatography/Mass Spectrometry and Gas Chromatography/Tandem Mass Spectrometry for Ambient Nitro- and Oxy-PAHs and Its Applications
Source: Molecules. 2023 Jan 12;28(2):775. doi: 10.3390/molecules28020775 (PMC9867094; doi:10.3390/molecules28020775)
Supplement: Supplementary file 1 [file molecules-28-00775-s001.zip › molecules-2064757-supplementary.pdf]

- Supplementary Material –

Development of Quantitative Chemical Ionization Using Gas Chromatography/Mass Spectrometry and Gas chromatography/Tandem Mass Spectrometry for Ambient Nitro- and Oxy-PAHs and Its Applications

Jungmin Jo [1], Ji Yi Lee [1], Kyoung-Soon Jang [2], Atsushi Matsuki[3], Amgalan Natsagdorj[4] and Yun Gyong Ahn [5,*]

[1] Dept. of Environmental Science and Engineering, Ewha Womans University, Seoul, Republic of Korea

[2] Bio-Chemical Analysis Team, Korea Basic Science Institute, Cheongju, 28119, Republic of Korea

[3] Institute of Nature and Environmental Technology, Kanazawa University, Kanazawa, Japan

[4] Department of Chemistry, National University of Mongolia, Ulaanbaatar, Mongolia

[5] Western Seoul Center, Korea Basic Science Institute, University-Industry Cooperation Building, 150, Bugahyeon-ro, Seodaemun-gu, Seoul, 03759, Republic of Korea

* Correspondence: ygahn@kbsi.re.kr

Contents:

Table S1. Comparison of molecular ion intensities of NPAHs when using EI and NCI modes at the concentration of 5 μg/mL

Table S2. Regional concentration range (ng/m^3), mean value and detection frequency of NPAH and OPAH congener in $PM_{2.5}$

Table S3. List of target compounds

Table S4. The analytical conditions of GC/MS and GC-QqQ (MS/MS)

Figure S1. Map of sampling locations

Table S1. Comparison of molecular ion intensities of NPAHs when using EI and NCI modes at the concentration of 5 μg/mL

NPAHs	MW	EI method		NCI method		NCI/EI (Intensity ratio)
		Intensity	Relative Intensity (%)	Intensity	Relative Intensity (%)	
1-NNAP	173	12831	42.3	195712	100.0	15
2-NNAP	173	22432	52.8	145792	100.0	7
2-NFLUO	211	34080	19.2	108232	100.0	3
9-NANT	223	47856	75.3	121888	100.0	3
3-NFL	247	86048	100.0	290112	100.0	3
4-NPYR	247	98656	68.5	340544	100.0	4
1-NPYR	247	92128	70.1	285312	100.0	3
6-NCHR	273	87728	75.3	304512	100.0	4

Table S2. Regional concentration range (ng/m³), mean value and detection frequency of NPAH and OPAH congener in $PM_{2.5}$

Type	Compound	Noto (n=28)		Seoul (n=31)		Ulaanbaatar (n=25)	
		Mean (range)	DF[a] (%)	Mean (range)	DF (%)	Mean (range)	DF (%)
NPAHs							
	1-NNAP	NA[b]	ND[c]	0.064 (0.061-0.070)	100	0.572 (ND-0.932)	96
	2-NNAP	NA	ND	0.167 (0.164-0.183)	100	1.022 (0.219-1.705)	100
	2-NFLUO	NA	ND	0.034 (0.022-0.057)	100	0.298 (0.152-0.677)	100
	9-NANT	NA	ND	0.316 (0.274-0.387)	100	0.600 (ND-2.139)	64
	3-NFL	0.001 (ND-0.031)	4	0.047 (0.026-0.111)	100	0.252 (ND-3.659)	76
	4-NPYR	NA	ND	0.037 (0.035-0.040)	100	0.117 (ND-0.255)	88
	1-NPYR	NA	ND	0.026 (0.018-0.048)	100	0.194 (0.083-0.352)	100
	6-NCHR	0.001 (ND-0.016)	4	NA	ND	NA	ND
	Total NPAHs	0.002 (ND-0.031)		0.6908 (0.618-0.832)		2.957 (1.099-7.627)	
OPAHs							
	9-Flu	0.184 (0.047-0.580)	100	1.967 (0.751-4.156)	100	75.279 (16.703-201.704)	100
	XT	0.021 (ND-0.298)	7	0.637 (0.463-0.802)	100	11.095 (2.600-23.006)	100
	PH	0.025 (ND-0.160)	71	1.093 (0.507-1.684)	100	63.212 (12.549-174.388)	100
	Anq	0.153 (0.013-0.629)	100	2.165 (1.187-3.414)	100	36.577 (10.632-78.043)	100
	1,8-NA	0.453 (0.002-3.175)	100	6.039 (3.833-10.131)	100	38.702 (12.024-91.782)	100
	2-Maq	0.005 (ND-0.129)	4	0.516 (0.364-0.641)	100	9.565 (3.688-21.137)	100
	BbFLU	0.010 (ND-0.082)	43	0.620 (0.516-0.788)	100	22.592 (5.604-51.449)	100
	BZA	0.021 (ND-0.106)	39	0.739 (0.600-1.008)	100	38.751 (9.956-91.946)	100
	BAQ	0.012 (ND-0.334)	4	0.444 (0.334-0.563)	100	9.147 (2.794-18.338)	100
	Ncq	NA	ND	0.483 (ND-0.600)	90	10.926 (4.722-25.270)	100
	Total OPAHs	0.884 (0.068-4.384)		14.703 (9.303-21.619)		315.846 (81.271-749.360)	

[a]DF : detection frequency , [b]ND : not detected , [c]NA : not applicate

Table S3. List of target compounds

Compound	Abbreviation	CAS number	Molecular	MW
Nitro-PAHs (NPAHs)				
1-Nitronaphthalene	1-NNAP	86-57-7	$C_{10}H_7NO_2$	173.17
2-Nitronaphthalene	2-NNAP	581-89-5	$C_{10}H_7NO_2$	173.17
2-Nitrofluorene	2-NFLUO	607-57-8	$C_{13}H_9NO_2$	211.22
9-Nitroanthracene	9-NANT	602-60-8	$C_{14}H_9NO_2$	223.23
3-Nitrofluoranthene	3-NFL	892-21-7	$C_{16}H_9NO_2$	247.25
4-Nitropyrene	4-NPYR	57835-92-4	$C_{16}H_9NO_2$	247.25
1-Nitropyrene	1-NPYR	5522-43-0	$C_{16}H_9NO_2$	247.25
6-Nitrochrysene	6-NCHR	7496-02-08	$C_{18}H_{11}NO_2$	273.29
Oxy-PAHs (OPAHs)				
9-Fluorenone	9-Flu	486-25-9	$C_{13}H_8O$	180.21
Phenalenone (Perinaphthenone)	PH	548-39-0	$C_{13}H_8O$	180.20
Xanthone	XT	90-47-1	$C_{13}H_8O_2$	196.20
1,8-Naphthalic anhydride	1,8-NA	81-84-5	$C_{12}H_6O_3$	198.17
Anthraquinone (9,10-Antraquinone)	Anq	84-65-1	$C_{14}H_8O_2$	208.21
2-Methylanthraquinone	2-Maq	84-54-8	$C_{15}H_{10}O_2$	222.24
7H-Benz[de]anthracene-7-one (benzoanthrone)	BZA	82-05-3	$C_{17}H_{10}O$	230.26
Benzo[b]fluoren-11-one	BbFLU	3074-03-1	$C_{17}H_{10}O$	230.26
5,12-Naphthacenequinone	Ncq	1090-13-7	$C_{18}H_{10}O_2$	258.27
Benz[a]anthracene-1,2-quinone	BAQ	2498-66-0	$C_{18}H_{10}O_2$	258.27
Internal standard				
Fluoranthene-d10	Fla-d10	9351-69-0	$C_{16}D_{10}$	212.14

Table S4. The analytical conditions of GC/MS and GC-QqQ (MS/MS)

Instrument	GC/MS<br>Agilent 7890B Gas Chromatograph<br>5977A MSD	GC-QqQ<br>Agilent 7890B Gas Chromatograph<br>7010 Triple Quad MSD
Column	DB-5MS UI (30m × 0.25mm × 0.25μm)	DB-5MS UI (60m × 0.25mm × 0.25μm)
Gas (gas flow)	CH_4 (CI reagent gas, 2mL/min)	N_2 (collision gas)<br>CH_4 (CI reagent gas, 1mL/min)
Injector (mode/Temp/Vol)	Splitless mode / 300 ˚C / 2uL	Splitless mode / 300 ˚C / 2uL
Ionization	NCI mode : 300 ˚C (ion source Temp.)	PCI mode : 300 ˚C (ion source Temp.)
Quantification	SIM mode	SRM mode
Oven program	60°C → 145°C (10°C/min) → 220°C (4°C/min) → 320°C (10°C/min, hold for 20 min)	

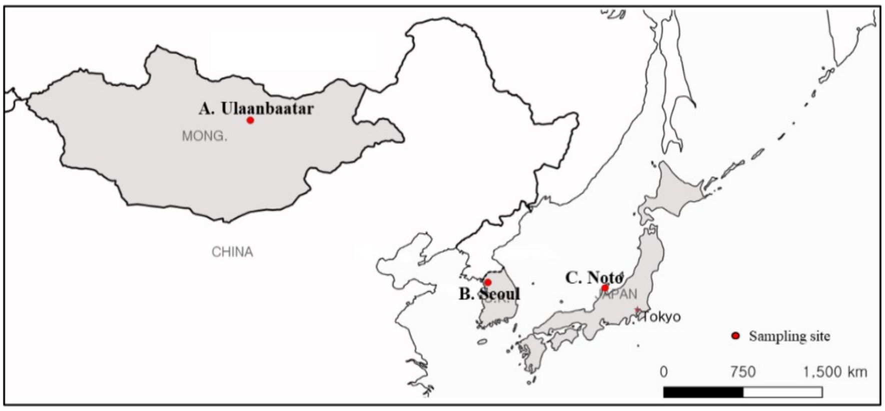

Figure S1. Map of sampling locations
